# Overuse of colorectal cancer screening services in the United States and its implications

**DOI:** 10.1186/s40880-016-0148-5

**Published:** 2016-09-15

**Authors:** John Bian

**Affiliations:** Department of Clinical Pharmacy and Outcomes Sciences, South Carolina College of Pharmacy, Columbia, SC 29208 USA

**Keywords:** Colorectal cancer, Screening services, Colonoscopy

## Abstract

As a standard way for prevention and early detection of colorectal cancer (CRC), colonoscopy has been used for CRC screening in the United States for more than one decade. An article entitled “Assessing Colorectal Cancer Screening Adherence of Medicare Fee-For-Service Beneficiaries Age 76 to 95 Years” recently published at the *Journal of Oncology Practice* reports the trends in overuse of CRC screening services among average-risk elderly populations at the age of 76–95 years. Several reasons for overusing colonoscopy have been postulated, and some strategies for reducing overuse of CRC screening services have also been proposed.

A few days ago, I ran into a middle-aged lady whom I used to see frequently jogging in our neighborhood. She appeared stressed out and very tired. I was concerned and inquired about how had been with her. She told me that she had been looking after her sick mother in the past 4 months. Her mother, in her mid-eighties, had been very healthy until she was hospitalized for pneumonia last December in 2015. During her hospitalization, her doctor seemingly randomly asked whether she ever had colorectal cancer (CRC) screening tests. After told no test being done in the past, her doctor ordered a screening colonoscopy while she was in the hospital. Unfortunately, this procedure led to perforation and forced her into a prolonged hospital stay. Her mother was still recovering from this colonoscopy-related complication.

An article entitled “Assessing Colorectal Cancer Screening Adherence of Medicare Fee-For-Service Beneficiaries Age 76 to 95 Years” recently published at the *Journal of Oncology Practice* (*JOP*) by Bian et al. [1] highlighted the trends in the overuse of CRC screening services among average-risk elderly populations at the age of 76–95 years from 2002 to 2010. CRC screening is an effective and a cost-effective way for reducing CRC-related death. However, these screening services, particularly colonoscopy, may carry some risks. Specifically, screening colonoscopies may expose patients to polypectomy-related perforation and bleeding risks and to sedation-related cardiovascular and pulmonary complications. The level of these risks may also be elevated with age. As a result, after weighing in the benefit-and-risk tradeoff, the United States (US) Preventive Services Task Force (USPSTF) does not recommend routine CRC screening to individuals over age 75 years at an average risk of CRC (e.g., without family history of CRC or prior polyp/adenoma history) [2]. In spite of the above explicit recommendation by the USPSTF, almost all major health insurance programs in the US, such as the Medicare program (a nationwide health insurance for the elderly age over 65 years), have continued their coverage for CRC screening of the average-risk individuals without setting upper-age limit. Consequently, there has been a growing concern about potential overuse of CRC screening services after Medicare started coverage of all four main CRC screening modalities (including colonoscopy) in 2001 [3].

This *JOP* article [1] reports an observational study designed for addressing this concern. This study used a 5% random non-cancer sample of Medicare fee-for-service (FFS) enrollees during 2002–2010 residing in the Surveillance, Epidemiology, and End Results (SEER) areas to construct a 9-year study cohort that included average-risk enrollees at the age of 76–95 years. The two outcomes of interest were the up-to-date status (in a given year) of adherence to overall CRC screening and to colonoscopy (versus the other three modalities). The authors additionally analyzed a sample of average-risk enrollees at the age of 86–95 years, in which any screening services received may be deemed overuse of CRC screening services according to the USPSTF recommendation.

The authors found that overall CRC screening adherence rates for studied individuals at the age of 76–95 years rose from 13.0% in 2002 to 21.4% in 2010. In 2002, 2.2% were adherent to colonoscopy, and 10.7% to the other three modalities; in 2010, the corresponding rates were 19.5 and 1.9%. The rapid rise in overall adherence rates may be a result of Medicare coverage policy of screening colonoscopy for average-risk beneficiaries starting in 2001, and a longer recommended screening interval of 10 years. Our additional analysis of the enrollees at the age of 86–95 years demonstrated that the overall adherence rates were almost doubled from 6.7% in 2002 to 12.5% in 2010. These increases, largely driven by colonoscopy use, may represent a significant portion of overuse of CRC screening services.

There are at least three plausible reasons for the observed potential overuse. First, the direct financial incentive in a Medicare FFS environment may promote over-prescription of these services. Second, because the CRC screening adherence rate has been widely used as a quality indicator for measuring performance of healthcare provider, perceived higher rates may suggest higher health care quality and in turn may indirectly lead to higher payment for better performance. Third, because many providers may be overwhelmed by day-to-day busy clinical practice, particularly in primary care settings, they have little time for consulting patients about the benefits and risks of various cancer preventive services such as CRC screening. In the end, the authors cautiously offered some corresponding remedies for reducing overuse of CRC screening services: redesigning value-based payment systems, measuring age-weighted CRC screening performance, and reimbursing physicians for their time discussing the trade-off of benefits and risks of screening colonoscopy.

Finally, this *JOP* article [1] may offer some insights of using observational data for cancer health services/outcomes research in China. The dataset used in this study was longitudinal non-cancer data, paralleled to the SEER data (the cancer registry data covering a quarter of the US populations). A large body of evidence has been published from these datasets on cancer topics related to epidemiology, prevention, treatment, and survivorship. In addition, these datasets have been increasingly used for cancer health policy research [4, 5]. Undoubtedly, the evidence generated from these valuable datasets may have contributed to remarkable improvement in cancer survivorship in the US over the past four decades. As cancer death is becoming a major health burden in China [6, 7], more research that uses nationally representative cancer registry and other observational data is critically needed to monitor progresses of quality and outcomes of cancer care in China.
